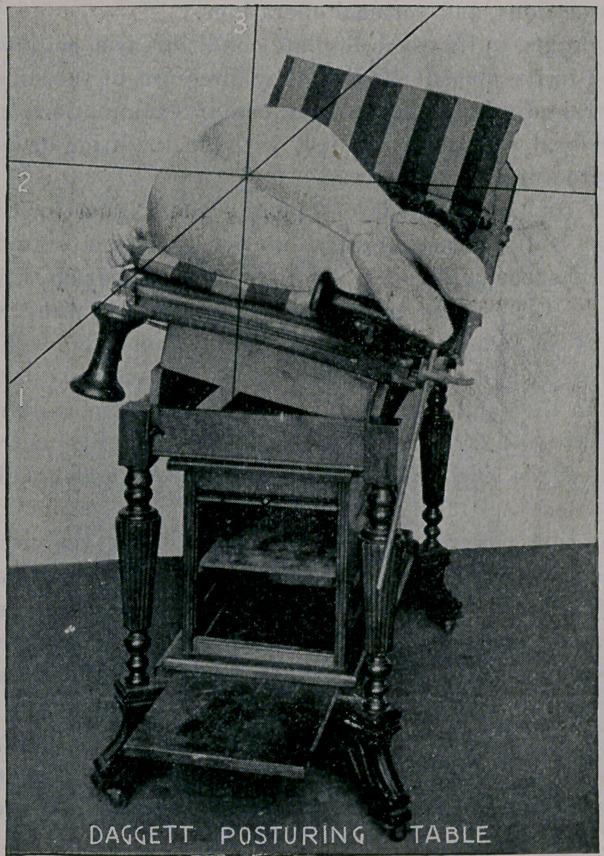# Concerning Posture1Copyrighted.

**Published:** 1893-09

**Authors:** B. H. Daggett

**Affiliations:** 258 Franklin Street; Buffalo


### CONCERNING POSTURE.[1]

By B. H. DAGGETT, M. D., Buffalo.

T. Lauder Brunton contributes to the November number of the *Popular Science Monthly*, an interesting article concerning posture and its indications, in which he discusses the varying attitudes and common postures one meets with daily; illustrates these postures in schematic outline, and inquires why they are assumed.

The variable attitudes of the body are attributed to trade, habit, mutable mental states, physiological activity, and pathological conditions.

Gravity in the upright posture is undoubtedly a contributing factor to engorgement of the pelvic viscera, rectal, uterine, and vaginal prolapse, varicose veins, hernia, and as we grow older to sagging of the bladder, and bagging of the rectum.

Our progenitors, moving about upon all fours, escaped, we presume, these direful calamities, and in due progress of evolution acquired the upright posture; eliminated the caudal appendage; consigned the appendix vermiformis to waste and final obliteration; and will, in due course, reinforce veinous valvular construction and develop complete support for the pelvic organs.

In the meantime we are called upon to alleviate conditions which confront and study theories which perplex.

It is proposed to consider, herein, some of the objective relations of this subject so far as they concern posturing for examination, treatment, and operation.

The varying attitudes of the body, the movements of the extremities, functional activity of the internal organs, and pathological conditions, modify in a greater or less degree the environment of the viscera, which should be taken into account in examination and operation.

Posture changes the site of the impulse of the normal heart, yet the text-books scarcely make reference to it.

Paul says that the patient may sit, stand upright, or recline for examination of the heart, as is most convenient.

Guttman alleges that the cardiac sounds are the loudest in the upright posture.

Dr. Azoulay, of Paris, claims that he has devised a means of intensifying the cardiac sounds by placing his patient upon the back, elevating the arms, flexing the lower extremities, and rising the

1. Copyrighted.

head, which reinforces the heart sounds and slows its action. Drs. Azoulay and Jules Simon have employed this method in the Children's Hospital, in Paris, and state that they have been able to localize extra- and intra-cardiac bruits, as the slowing of the heart's action and the augmentation of its sounds aided in clearing up their vagueness and complicated character.

It is said that dyspnea and arythmia without slowing of the

DAGGETT POSTURING TABLE

pulse, in the recumbent posture, and which do not occur in the upright, afflicting a person supposed to be in good health, indicate a lesion of the myocardium, and a fatal issue from asytole may be predicted at an early date, according to the degree of the disease.

If this be true, applicants for life insurance should be postured for examination; indeed, in all obscure cases diagnosticians could profitably utilize natural attitudes and assumed postures when conducting physical examinations.

Sims discovered and demonstrated the advantages of the side posture; still, with all that has been said and written, a comparatively small number of physicians employ it.

Many specialists use the side position for ocular examination, and treatment; the dorsal recumbent posture for executing bimanual examinations, and perineal operations.

The vagina, in its usual desolate condition, is a collapsed sack, preserved in its closed condition by pressure of the superincumbent, surrounding viscera, so that ocular examination cannot be made, or local treatment applied, without dilatation or expansion of this structure.

There are two methods of opening the vagina for these purposes, one is direct mechanical dilatation by means of cylindrical or valvular specula, the other is by elevation of the pelvis to induce prolapse of the viscera toward the diaphragm by the traction of gravity, at the same time retracting the perineum.

Dr. Joseph Price declares the cylindrical and valvular specula do harm in the hands of the inexpert, and the expert do not need them; that if ocular examination is necessary, or local treatment is indicated, the side position and Sims' speculum should always be used.

In the side position upon a horizontal plane the viscera still press upon the vagina, so that traction upon the perineum and repression upon the anterior surface of the vagina are necessary to provide a free field for the purposes of inspection and treatment. To carry out these manipulations requires the aid of a skilled assistant or a deft operator.

This obstacle is overcome by placing the patient in *the knee-thigh-chest posture*, described in the following paragraphs:

This position is not uncomfortable, and it combines the advantages of both the knee-chest and side (Sims') postures; it may be called the exaggerated Sims.

*The knee-thigh-chest posture.*—The patient mounts the step with the left thigh towards the table, lifts her drapery upon the table's top, in order that it may not bind her limbs as she reclines, or obstruct the operator. Reclining, she rests her left thigh across the table's end, carries her left arm backward, parallel with the body, places her left ankle upon the rest and draws the right limb over and beyond its fellow. She will posture herself unaided after one or two lessons.

The arms should not be carried upward, as this movement draws upon the thorax, causing tension of the abdominal muscles. The head is placed upon a pillow, a little beyond the middle line, so that the body is slightly flexed, relaxing the abdominal muscles. The left thigh and leg are flexed to, or slightly beyond, right

DAGGETT POSTURING TABLE

angles, and the right are more fully flexed, the knee dipping downward. These flexures relax the pelvic muscles.

In this position, upon a horizontal plane, it is evident there is very little dip to the abdominal cavity, and its viscera moving by respiration in a horizontal line press upon the pelvic organs.

To avoid this, the table top is given a double tilt. The high lateral tilt inclines the body so that the patient turns upon the left thorax, and the knees rest upon the side rail, the longitudinal tilt accentuates this position, which gives a decided dip to the long diameter of the abdominal cavity, and the viscera settle towards the diaphragm.

For this position, the side tilt should be raised to an angle of twelve or more degrees from the horizontal line ; the long tilt to an angle of five or six degrees ; more than this for the long tilt renders the patient uncomfortable and is unnecessary.

The tilting is to be done after the patient is placed as described. A line drawn through the sacral plane is transversely with the body, at an angle of forty-five degrees ; lengthwise with the body, at an angle of six degrees from a perpendicular line.

Thomas made a study of this subject, and as a result of his experiments constructed the gynecological table which bears his name.

As bodies differ in conformation, set rules are modified in accordance with the tact and judgment of the operator.

The cut on the opposite page illustrates the knee-thigh-chest posture, in which it will be observed that the body is comfortably supported by the knees resting upon a wide side-rail.

258 Franklin Street.

## Figures and Tables

**Figure f1:**
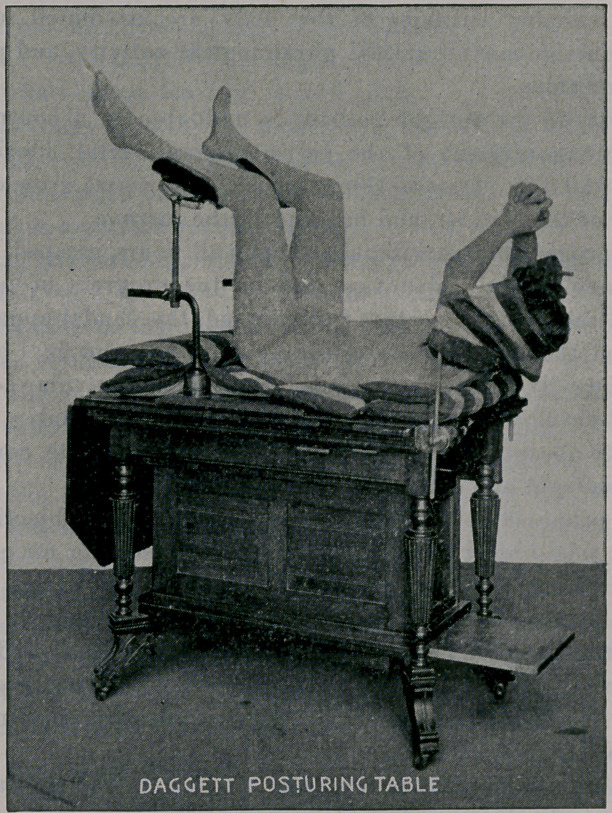


**Figure f2:**